# The mediation effect of contraceptive use and women’s autonomy on the relationship between intimate partner violence and unintended pregnancy in Ethiopia

**DOI:** 10.1186/s12889-020-09514-7

**Published:** 2020-09-16

**Authors:** Tenaw Yimer Tiruye, Melissa L. Harris, Catherine Chojenta, Elizabeth Holliday, Deborah Loxton

**Affiliations:** 1 Public health department, College of Health Sciences, Debre Markos University, Debre Markos, Ethiopia; 2 Research Centre for Generational Health and Ageing, School of Medicine and Public Health, Faculty of Health and Medicine, the University of Newcastle, Newcastle, Australia; 3 School of Medicine and Public Health, Faculty of Health and Medicine, University of Newcastle, Newcastle, Australia

**Keywords:** Intimate partner violence, Unintended pregnancy, Women’s autonomy, Contraception use, Mediation analysis, Demographic and health survey, Ethiopia

## Abstract

**Background:**

Intimate partner violence (IPV) affects one in every three women globally. Previous studies have revealed that women’s experiences of different forms of IPV are significantly associated with a higher rate of unintended pregnancy, reduced uptake of contraception, and reduced ability to make decisions regarding their fertility. The aim of this study was to investigate whether previously observed relationships between IPV and unintended pregnancy in Ethiopia are mediated by contraceptive use and women’s autonomy.

**Methods:**

This study was performed using nationally representative data from the 2016 Ethiopian Demographic and Health Survey (EDHS). A subsample of married women of reproductive age reporting a pregnancy within the 5 years preceding 2016 and who participated in the domestic violence sub-study of the survey were included in analyses. Logistic regression models, together with the product of coefficients method, were used to estimate direct and mediated effects.

**Results:**

Twenty six percent of participants reported an unintended pregnancy in the 5 years preceding the survey. Sixty-four percent reported having ever experienced IPV (a composite measure of physical, sexual, emotional abuse, and partner controlling behaviour). After adjusting for potential confounding factors, unintended pregnancy was significantly positively associated with reporting sexual IPV, emotional IPV, IPV (a composite measure of physical, sexual, or emotional abuse), and multiple partner controlling behaviour. However, IPV (as a composite of all four forms), physical IPV, and partner control (single act) were not significantly associated with unintended pregnancy. Women’s autonomy, but not contraception use, had a significant partial mediation effect in the relationships between some forms of IPV and unintended pregnancy. Women’s autonomy mediated about 35, 35, and 43% of the total effect of emotional IPV, IPV (physical, sexual, and/or emotional), and multiple partner control on unintended pregnancy respectively.

**Conclusion:**

Women’s autonomy appears to play a significant role in mediating the effect of IPV on unintended pregnancy in Ethiopia. Maternal health service interventions in Ethiopia could incorporate measures to improve women’s decision-making power to reduce the negative reproductive health effects of IPV.

## Background

Intimate partner violence (IPV) includes acts of physical aggression, psychological abuse, sexual coercion and controlling behaviours within an intimate relationship [1]. IPV affects one in every three women globally [2]. It has several effects on women’s physical, mental, and reproductive health [3–5]. The reproductive health consequences of IPV include sexually transmitted infections [6–8], obstetric complications such as haemorrhage, abortion, hypertensive disorders, and foetal complications [3, 9], and reduced utilization of maternal health services [4, 10].

Studies have revealed that women’s experiences of different forms of IPV are significantly associated with a higher rate of unintended pregnancy [11–17], defined as pregnancies that are either unwanted or mistimed. Other studies have also demonstrated that IPV is associated with reduced uptake of contraception [18–23]. Women who report abuse are also more likely to have partners that make decisions for them about contraception and whether and when to have a baby [24–27]. This implies that women experiencing IPV have reduced ability to use contraception and make decisions regarding their fertility, both of which could negatively affect their ability to enact their reproductive intentions.

There is evidence that unintended pregnancy is mainly the result of inadequate contraception practice such as incorrect/non-use of contraception, discontinuation of contraceptives, and contraceptive failure [28–31]. Researchers also identified that low women’s autonomy is a significant predictor of unintended pregnancy [32–34]. Women’s autonomy relates to women’s power and ability to control over resources, making their own decisions, improve and maintain their health, and seek necessary information for their reproductive choices [32]. There is recognition of the importance of the interplay between IPV with contraception use, IPV with women’s autonomy, IPV with unintended pregnancy, contraception use with unintended pregnancy, and women’s autonomy with unintended pregnancy. However, there is limited evidence on the pathways by which the four experiences are inter-related i.e. how IPV affects unintended pregnancy through contraception use and women’s autonomy is less known. Furthermore, some researchers [11, 14, 35] who investigated the relationship between IPV and unintended pregnancy have treated the contraception use variable as a confounder; they explain that IPV might affect unintended pregnancy by affecting women’s contraception use. However, we argue that in the interplay between these three experiences, contraception use should be considered as a mediator rather than a confounder. In mediation, a third variable (the mediator) partly conveys the causal relationship between the exposure and outcome (Exposure → Mediator → Outcome). In contrast, with confounding a third variable (the confounder) causally affects both the exposure and the outcome. Adjustment for confounders is necessary to estimate unbiased causal effects, but confounders do not convey the causal relationship among the exposure and outcome [36].

The current study is from Ethiopia, which is generally characterized by high gender inequality, high fertility (total fertility rate of 4.6 children per woman) [37], low contraception use (only 36% of women use modern contraception) [37], high rates of unintended pregnancy (25%) [37], and one of the highest national rates of IPV (ranging from 20 to 78% in different areas of the country) [38]. In the current analysis, we hypothesized that women’s lifetime experience of IPV would be associated with contraception use and women’s autonomy in a sample of married Ethiopian women. While IPV may affect unintended pregnancy, it is likely that this effect would be mediated by contraception use and women’s autonomy that are influenced by IPV and affect unintended pregnancy.

Most available literature on IPV and its negative effects preclude defining partner controlling behaviour as a form of IPV, and consider IPV as a composite measure of physical, sexual, and emotional abuse. However, there is evidence that partner controlling behaviour is a reflection of power dynamics in an intimate relationship and indicates imminent risk of other forms of abuse [27, 39]. Studies have also revealed that partners’ control influences women’s decision-making power [27], health service access and utilization [27, 40], and fertility control [15, 41, 42]. Therefore, we have adopted the World Health Organization (WHO) definition of IPV (1 p89) and included partner control as a form of IPV in our analysis.

The hypotheses tested in this study were:

1. Women who have experienced any form of IPV are more likely to have higher odds of unintended pregnancy than women who have not experienced IPV.
2. Composite measures of IPV are positively associated with unintended pregnancy.
3. Contraception use and women’s autonomy mediate the impact of IPV on unintended pregnancy.
4. The concentration of partner controlling behaviours has a more significant effect on the mediators and the outcome than any single behaviour.

## Methods

### Data source, design and population

This study used data from the 2016 Ethiopian Demographic and Health Survey (EDHS), which was a cross-sectional national survey conducted from 18 January to 27 June 2016. In total, 15,683 women aged 15–49 years were sampled using random selection. For the domestic violence sub-study, only one married woman per household was selected and 5860 women were interviewed [37]. Due to the complex sampling procedures (multi stage stratified cluster sampling) used by the EDHS, sampling weights were adjusted for differences in probability of selection that allow extrapolation of results to the national level of representativeness [37].

### Sample size

**Fig. 1:**
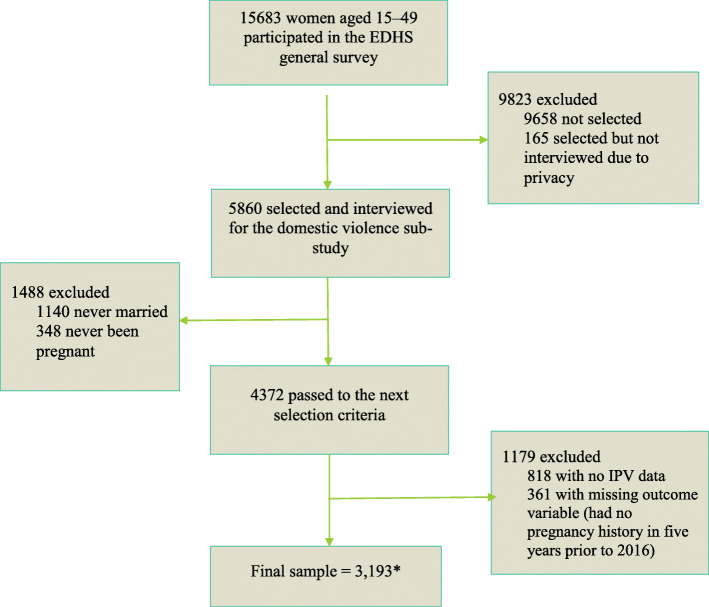
Schematic representation of participant selection procedure Key: EDHS, Ethiopian Demographic and Health Survey; IPV, Intimate Partner Violence; *the final sample shown is unweighted and the weighted sample, which is reported throughout the paper, is 2,969

For this analysis, 2969 (weighted) married women who had been pregnant within 5 years preceding 2016, who had complete data related to their reproductive intentions and responded to the IPV questionnaire were included. For mothers with more than one pregnancy, we used the most recent pregnancy for the study (Fig. 1).

### Measurement and variables

#### Dependent variable

In the woman’s questionnaire of the EDHS, the reproductive intentions of the women about each pregnancy and/or birth within the past 5 years were measured by asking participants to recall their feelings at the time of pregnancy. The optional answers were: wanted at that time of pregnancy (planned), wanted the pregnancy to happen later (mistimed), and did not want the pregnancy at all (unwanted). For the purposes of this analysis, an unintended pregnancy was defined as a pregnancy in the past 5 years that was either mistimed (i.e. the pregnancy was wanted but occurred earlier (within 2 years) than expected) or unwanted [37]. As such, the pregnancy intention of participants was categorized as unintended or intended.

#### Exposure variable

In the EDHS, women were asked whether or not they had experienced violent and controlling acts within their relationship, perpetrated by their husband/partner for currently married women and recent husband/partner for previously married women. Respondents were categorized as having experienced lifetime IPV if they reported experiencing at least one act of IPV [37]. Table 1 presents the questions used to assess IPV and the form of IPV the questions measuring. To further test the hypothesis that the concentrations of behaviours have a more significant effect than any single behaviour on unintended pregnancy, we recoded the partner controlling behaviour variable into none, single act, and multiple acts (where women reported two or more acts of partner controlling behaviour). We have also further investigated IPV as a composite measure of physical, sexual, and emotional abuse to allow comparison with previous research following a similar approach.

**Table 1:** The tool used to assess IPV in the 2016 Ethiopian Demographic and Health Survey

IPV type	Question/item^a^
Physical IPV	Push you, shake you, or throw something at you?
	Slap you?
	Twist your arm or pull your hair?
	Punch you with his/her fist or with something that could hurt you?
	Kick you, drag you, or beat you up?
	Try to choke you or burn you on purpose?
	Threaten or attack you with a knife, gun, or any other weapon?
Sexual IPV	Physically force you to have sexual intercourse with him even when you did not want to?
	Physically force you to perform any other sexual acts you did not want to?
	Force you with threats or in any other way to perform sexual acts you did not want to?
Emotional IPV	Say or do something to humiliate you in front of others?
	Threaten to hurt or harm you or someone close to you?
	Insult you or make you feel bad about yourself?
Partner controlling behaviour	Being jealous if you talk to men?
	Accusing you of being unfaithful?
	Does not allow you to meet your friends?
	Limits you contact with family?
	Tries to know where you are at all times?

*IPV* Intimate partner violence; ^a^those women who were married more than once were further asked about violence committed by any other husband/partner

#### Mediator variables

Two variables − women’s autonomy and contraceptive use before the pregnancy − were considered as potential mediators. The EDHS questionnaire asked about women’s autonomy in decision-making regarding her own health care, major household purchases, and visits to her family or relatives. Women’s autonomy was coded as ‘yes’ if women reported being involved in all the three decisions, either alone or with partner or with any other person (in which she has a say in the decisions) [37]. The contraceptive use variable, which was women’s contraceptive use status before the pregnancy, was extracted from the contraceptive calendar data based on the Demographic and Health Survey (DHS) contraceptive calendar guide [43]. Then, contraceptive use variable was grouped into ‘yes’ if women used any method of contraception prior to the most recent pregnancy and ‘no’ if women didn’t use any contraception.

#### Covariates

Seven potentially confounding variables were identified based on prior knowledge [11–13, 35, 44] and context. Accordingly, current age of the respondent (15–19/20–24/25–29/30–34/35–39/40–44/45–49 years), respondent’s educational status (No formal education /primary/secondary+), religion (Christian/Muslim/other), rurality (urban/rural), region (11 administrative regions), number of children ever born (≤ 1/2 − 3/ ≥ 4), and wealth index were controlled for in the final analysis. Household wealth index was measured based on the number and kind of goods households had and housing characteristics (drinking water, toilet facility, flooring material and availability of electricity), and was generated using principal component analysis and classified into quintiles from 1 (very poor) to 5 (very rich) [37]. The DHS standard recode manual was used to define and code variables [45].

### Statistical analysis

Univariate descriptive statistics were calculated for variables. Chi-square statistics were calculated to compare IPV experience and participant characteristics by pregnancy intention. Survey data analysis techniques with Stata’s ‘*svy’* command were used throughout the analysis to account for complex survey data. All the analyses was conducted using Stata version 15.0 [46].

The analysis involved two steps. First, we assessed the independent association of each IPV form with unintended pregnancy using logistic regression models, adjusting for potential confounders. Then, the IPV forms showing significant association with unintended pregnancy in step one were further analysed to assess whether the hypothesized mediators mediated the observed relationships using mediation analysis with multiple mediators. Mediation analysis helps to understand the mechanisms through which exposure variables affect dependent variables [47].

To evaluate the unadjusted associations between the exposure variables (different forms of IPV), potentially mediating factors (contraceptive use and women’s autonomy), and the outcome (unintended pregnancy), we first constructed initial path models with mediating variables using the Structural Equation Modelling (SEM) builder in Stata. Then, fully adjusted multivariate mediation models were constructed using the Stata ‘*gsem*’ command controlling for potential confounders. Exposure variables (except partner controlling behaviour which was a categorical variable: none, single and multiple acts), mediating factors and unintended pregnancy were modelled as a binary variable. Hence, the entire path models i.e. paths linking exposure variables to mediators (path a), paths linking mediators to the outcome (path b), and paths linking exposure variables to the outcome (path c) represented a logistic model.

The mediation analysis was conducted based on the Baron and Kenny (1986) approach of testing mediation [47]. Accordingly, bivariate associations were calculated along the three paths between the three variables: Path a, Path b, and Path c. Mediation exists when the outcome variable is simultaneously regressed onto the exposure and the mediator, i.e. controlled for paths a and b, the coefficient for path c (indicated by *c*′) is reduced in both magnitude and significance level. If path *c*′ is reduced to zero, this indicates full mediation. If path *c*′ is not reduced to zero but still reduced in both magnitude and significance level, this suggests partial mediation [47].

In addition, we employed the product of coefficients method to statistically test if the exposure variables indirectly affected unintended pregnancy through the mediators. In the products method, path a and path b coefficients are multiplied and divided by the product of their related standard errors [48]. The Stata ‘*nlcom*’ command was used to statistically evaluate this; significant result of the ‘indirect effects’ indicates that mediation exists. The ‘*nlcom*’ command also enables to estimate the direct effect (*path c*′), indirect (mediated) effects (*path a* ∗ *path b*), and total effect (*path c*^′^ + (*path a* ∗ *path b*)) of IPV and partner controlling behaviours on unintended pregnancy. We executed ‘*nlcom’* three times to estimate the indirect effects: once for each of the two specific indirect effects of the two mediators (contraceptive use and women’s autonomy) and once for the total indirect effect. Finally, the proportion of total effect that is mediated was calculated as $\frac{coefficient\ of\ indirect\ effect\ }{coefficient\ of\ total\ effect}\ast 100\%$[49].

## Results

### Participant characteristics

The mean age of respondents was 29 years (SD±7 years, range: 15–49 years). The majority of study participants had no formal education (62.9%), were Christian (61.0%) and living in a rural area (87.2%). In total, 36.4% of participants reported having no decision-making autonomy and 76.0% of participants reported not having used any form of contraceptive before the pregnancy. Additional characteristics of participants are shown in Table 2.

**Table 2:** Intimate partner violence experience and participant characteristics by pregnancy intention, Ethiopian Demographic and Health Survey, 2016

Variable	Class	Total weighted sample (*n* = 2969)	Pregnancy intention		
			Intended (*n* = 2181)	Unintended^a^ (*n* = 788)	*P*-Value*
		No (%)	No (%)	No (%)	
Physical IPV	No	2226 (75.0)	1659 (76.1)	567 (72.0)	0.143
	Yes	743 (25.0)	522 (23.9)	221 (28.0)	
Sexual IPV	No	2615 (88.1)	1959 (89.8)	656 (83.2)	0.001
	Yes	354 (11.9)	222 (10.2)	132 (16.8)	
Emotional IPV	No	2285 (77.0)	1723 (79.0)	562 (71.3)	0.006
	Yes	684 (23.0)	458 (21.0)	226 (28.7)	
Partner controlling	No	1293 (43.6)	979 (44.9)	314 (39.8)	0.114
	Yes	1676 (56.4)	1202 (55.1)	474 (60.2)	
IPV^b^	No	1909 (64.3)	1454 (66.6)	455 (57.7)	0.004
	Yes	1060 (35.7)	727 (33.4)	333 (42.3)	
IPV (all)^c^	No	1059 (35.7)	801 (36.7)	257 (32.7)	0.211
	Yes	1910 (64.3)	1380 (63.3)	531 (67.3)	
Contraception use	No	2256 (76.0)	1625 (74.5)	631 (80.1)	0.029
	Yes	713 (24.0)	556 (25.5)	157 (19.9)	
Decision making autonomy	No	1083 (36.5)	727 (33.3)	356 (45.2)	<0.001
	Yes	1886 (63.5)	1454 (66.7)	432 (54.8)	
Current age	15–19	158 (5.3)	120 (5.5)	38 (4.8)	0.007
	20–24	548 (18.5)	432 (19.8)	116 (14.7)	
	25–29	838 (28.2)	642 (29.4)	197 (25.0)	
	30–34	678 (22.9)	489 (22.4)	189 (24.0)	
	35–39	472 (15.9)	329 (15.1)	143 (18.2)	
	40–44	205 (6.9)	131 (6.0)	74 (9.4)	
	45–49	69 (2.3)	37 (1.7)	32 (4.1)	
Educational status	No formal education	1866 (62.9)	1372 (62.9)	494 (62.7)	0.536
	Primary	813 (27.4)	585 (26.8)	228 (29.0)	
	Secondary+	290 (9.8)	224 (10.3)	66 (8.4)	
Religion	Christian	1812 (61.0)	1335 (61.2)	477 (60.5)	0.730
	Muslim	1089 (36.7)	801 (36.7)	289 (36.7)	
	Other	68 (2.3)	45 (2.1)	22 (2.8)	
Rurality	Urban	379 (12.8)	287 (13.2)	92 (11.7)	0.475
	Rural	2589 (87.2)	1894 (86.8)	696 (88.3)	
Region of residence	Tigray	198 (6.7)	158 (7.2)	40 (5.1)	<0.001^¥^
	Afar	28 (0.9)	25 (1.1)	3 (0.4)	
	Amhara	666 (22.4)	494 (22.7)	172 (21.9)	
	Oromia	1222 (41.2)	869 (39.8)	353 (44.8)	
	Somali	104 (3.5)	101 (4.6)	3 (0.4)	
	Benishangul	31 (1.0)	26 (1.2)	5 (0.6)	
	SNNPR	629 (21.2)	444 (20.4)	185 (23.5)	
	Gambela	8 (0.3)	6 (0.3)	2 (0.2)	
	Harari	6 (0.2)	5 (0.2)	1 (0.1)	
	Addis Ababa	66 (2.2)	45 (2.1)	21 (2.6)	
	Dire Dawa	12 (0.4)	9 (0.4)	3 (0.4)	
Number of children ever born	One or less	606 (20.4)	486 (22.3)	120 (15.3)	<0.001
	Two-three	875 (29.5)	682 (31.2)	193 (24.5)	
	Four or more	1488 (50.1)	1014 (46.5)	474 (60.2)	
Wealth index	Poorest	633 (21.3)	476 (21.8)	157 (19.9)	0.181
	Poorer	638 (21.5)	449 (20.6)	189 (24.0)	
	Middle	658 (22.2)	506 (23.2)	152 (19.3)	
	Richer	550 (18.5)	383 (17.5)	167 (21.2)	
	Richest	490 (16.5)	367 (16.8)	123 (15.6)	

^a^The prevalence of unintended pregnancy was 788(26.5% (95%CI: 24.2–28.9%)); *P-value was based on chi-squared test; *IPV* intimate partner violence; ^b^‘Yes’ if women reported experiencing physical, sexual or emotional abuse; ^c^‘Yes’ if women reported experiencing at least one of the four IPV forms; *SNNPR* Southern Nations, Nationals and Peoples Region; ^¥^*P*-value was based on Fisher’s exact test. All the weighted numbers and percentages are rounded

About 26% of women reported that their last pregnancy was unintended and 64% of participants reported having ever experienced IPV (a composite measure of physical, sexual, emotional abuse, and partner controlling behaviour). The least prevalent form of IPV was sexual IPV, (*n* = 353 (11.9%)), and the most prevalent form was partner controlling behaviour, (*n* = 1675 (56.4%)) (Table 2).

In univariate analysis, compared to those who reported an intended pregnancy, women who reported an unintended pregnancy reported lower rates of decision-making autonomy (*p* < 0.001) and contraceptive use (*p* = 0.029), and a higher number of children (p < 0.001) (Table 2).

### The association of different forms of IPV with unintended pregnancy

In the unadjusted logistic model, a significant association was observed between unintended pregnancy and having experienced sexual IPV (Crude Odds Ratio (COR) 1.78, 95% CI: 1.26, 2.50), emotional IPV (COR 1.52, 95% CI: 1.13, 2.04), IPV (physical, sexual, and/or emotional) (COR 1.46, 95% CI: 1.13, 1.90), and having experienced multiple acts of partner control (COR 1.67, 95% CI: 1.24, 2.24). There was no significant association between unintended pregnancy and experiencing physical IPV (COR 1.23, 95% CI: 0.93, 1.64), a single act of partner control (COR 1.34, 95% CI: 0.99, 1.83), and IPV as a composite measure of all four forms (COR 1.20, 95% CI: 0.90, 1.59) (Table 3).

**Table 3:** Associations between different forms of intimate partner violence and unintended pregnancy, Ethiopian Demographic and Health Survey, 2016

IPV forms	Category	Participant had unintended pregnancy			
		COR (95% CI)	p-value	AOR (95% CI)^a^	*p*-value
Physical IPV	No	Reference			
	Yes	1.23 (0.93, 1.64)	0.144	1.19 (0.87, 1.62)	0.270
Sexual IPV	No	Reference			
	Yes	1.78 (1.26, 2.50)	0.001	1.71 (1.18, 2.48)	0.004
Emotional IPV	No	Reference			
	Yes	1.52 (1.13, 2.04)	0.006	1.40 (1.02, 1.93)	0.037
Partner controlling behaviour	No	Reference			
	Yes	1.23 (0.95, 1.59)	0.115	1.18 (0.91, 1.53)	0.210
IPV (all)^b^	No	Reference			
	Yes	1.20 (0.90, 1.59)	0.211	1.13 (0.85, 1.49)	0.406
IPV (physical, sexual, or emotional)^c^	No	Reference			
	Yes	1.46 (1.13, 1.90)	0.005	1.39 (1.05, 1.85)	0.021
Partner controlling behaviour^d^	No	Reference			
	Single	1.34 (0.99, 1.83)	0.062	1.30 (0.95, 1.79)	0.102
	Multiple	1.67 (1.24, 2.24)	0.001	1.57 (1.16, 2.14)	0.004

*IPV* intimate partner violence, *COR* crude odds ratio, *CI* confidence interval, *AOR* adjusted odds ratio; ^a^The models were adjusted for age of women, education, rurality (urban/rural), religion, region of residence, number of children ever born, and wealth; ^b^‘Yes’ if women reported experiencing at least one of the four IPV forms; ^c^we assessed this association to allow comparison with previous research that investigated IPV as a combination of physical, sexual, and emotional abuse only i.e. without including partner control; ^d^we tested the hypothesis that the concentration of behaviours (multiple controlling acts) have a more significant effect than any single behaviour on unintended pregnancy

After adjusting for potential confounders, the significant associations of unintended pregnancy with sexual IPV (Adjusted Odds Ratio (AOR) 1.71, 95% CI: 1.18, 2.48), emotional IPV (AOR 1.40, 95% CI: 1.02, 1.93), IPV (physical, sexual, and/or emotional) (AOR 1.39, 95% CI: 1.05, 1.85), and multiple partner control (AOR 1.57, 95% CI: 1.16, 2.14) persisted (Table 3).

### Mediation analysis results

**Fig. 2:**
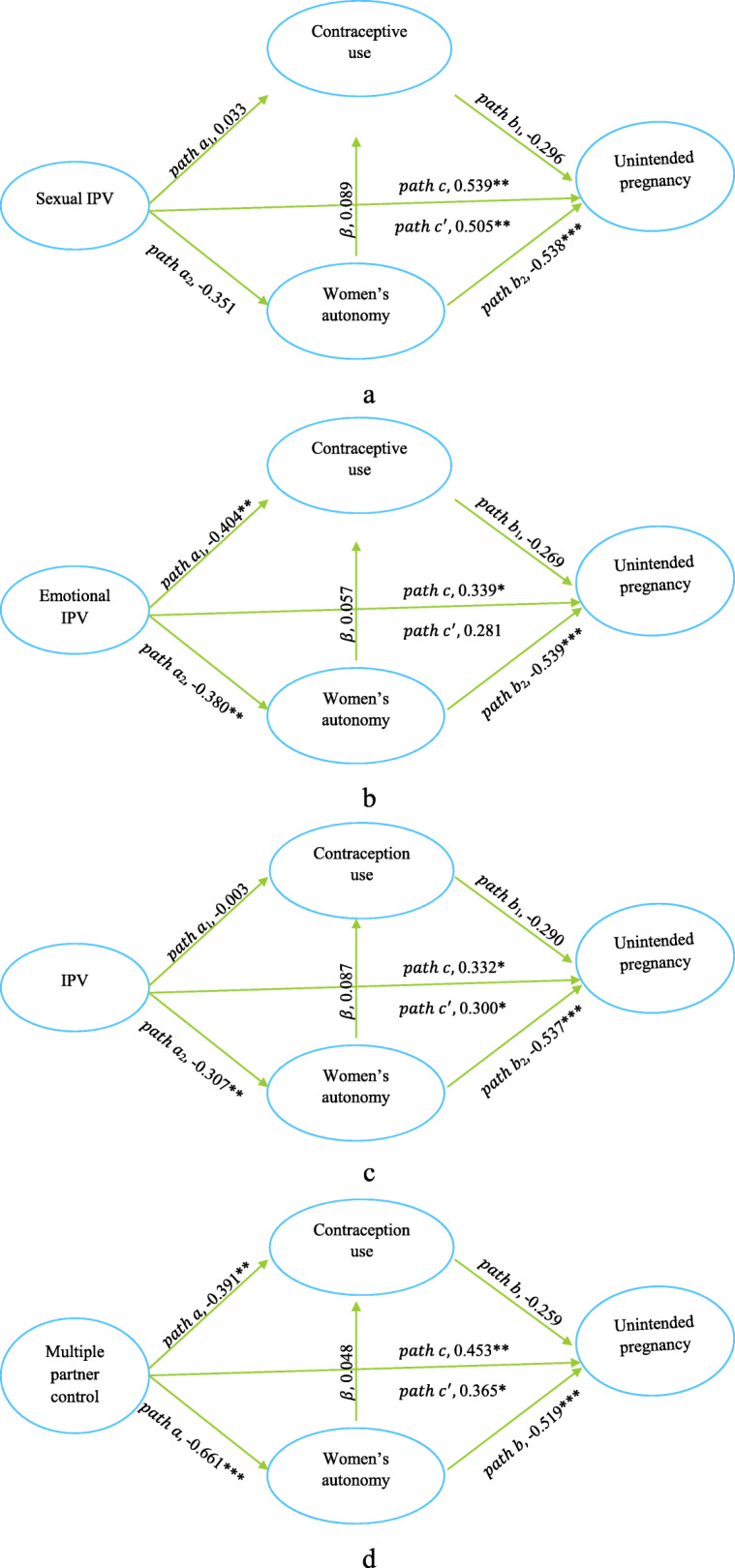
**a-d** Mediation effects for contraceptive use and women’s autonomy on the relationship between different forms of IPV and unintended pregnancy Key: Multiple regression coefficients determined by steps 1–3 of the mediation analysis are given along the path line. ***p < 0.001, **p < 0.01, *p < 0.05. All models were adjusted for age, number of children, education, religion, household wealth index, rurality, and region

In the first mediation model (Fig. 2a, Table 4), sexual IPV was significantly associated with unintended pregnancy (*path c*, *β* = 0.539, *p* = 0.004) but sexual IPV was not significantly associated with contraceptive use (*path a*_1_, *β* = 0.033, *p* = 0.872) nor with women’s autonomy (*path a*_2_, *β* =  − 0.351, *p* = 0.053). After controlling for contraception use and women’s autonomy, the coefficient for sexual IPV was decreased in magnitude and significance (p*ath c*^′^, *β* = 0.505, *p* = 0.008). However, as both *path a*’s in this model were not significant, neither mediator met established criteria for mediation.

**Table 4:** Associations between exposure, mediator and outcome variables, Ethiopian Demographic and Health Survey, 2016

Models	Associations between	*β*^a^	p-value
Sexual IPV^b^	Sexual IPV and contraception use	0.033	0.872
	Sexual IPV and women’s autonomy	−0.351	0.053
	Sexual IPV and unintended pregnancy	0.539	0.004
Emotional IPV	Emotional IPV and contraception use	−0.404	0.007
	Emotional IPV and women’s autonomy	−0.380	0.006
	Contraception use and unintended pregnancy^c^	−0.269	0.103
	Women’s autonomy and unintended pregnancy^e^	−0.539	<0.001
	Women’s autonomy and contraception use^d^	0.057	0.699
	Emotional IPV and unintended pregnancy	0.339	0.037
	Effect of emotional IPV on unintended pregnancy when controlling for contraception use and women’s autonomy	0.281	0.094
IPV (physical, sexual, emotional)	IPV and contraception use	−0.003	0.982
	IPV and women’s autonomy	−0.307	0.009
	Contraception use and unintended pregnancy^c^	−0.290	0.079
	Women’s autonomy and unintended pregnancy^e^	−0.537	<0.001
	Women’s autonomy and contraception use^d^	0.087	0.553
	IPV and unintended pregnancy	0.332	0.021
	Effect of IPV on unintended pregnancy when controlling for contraception use and women’s autonomy	0.300	0.040
Multiple partner control behaviours	Control (multiple) and contraception use	−0.391	0.006
	Control (multiple) and women’s autonomy	−0.661	<0.001
	Contraception use and unintended pregnancy^c^	−0.259	0.113
	Women’s autonomy and unintended pregnancy^e^	−0.519	<0.001
	Women’s autonomy and contraception use^d^	0.048	0.749
	Control (multiple) and unintended pregnancy	0.453	0.004
	Effect of multiple partner controlling acts on unintended pregnancy when adjusting for contraception use and women’s autonomy	0.365	0.022

^a^The models were adjusted for age of women, education, rurality (urban/rural), religion, region of residence, number of children ever born, and wealth; IPV, Intimate Partner Violence; ^b^ the remaining path coefficients of sexual IPV were not estimated because the first model to estimate *path a* were insignificant for both mediators; ^c,d,e^The associations were different in the succeeding models because the models were adjusted for different forms of IPV

In the second mediation model (Fig. 2b, Table 4), emotional IPV was significantly associated with contraceptive use (*path a*_1_,* β* =  − 0.404, *p* = 0.007), women’s decision-making autonomy (*path a*_2_, *β* =  − 0.380, *p* = 0.006), and unintended pregnancy (*path c*, *β* = 0.339, *p* = 0.037). The association between women’s autonomy and unintended pregnancy, controlling for emotional IPV, was also significant (*Path b*_2_,* β* =  − 0.539, *p* < 0.001) but the association between contraceptive use and unintended pregnancy was not significant (*Path b*_1_,* β* =  − 0.269, *p* = 0.103). In addition, the path from women’s autonomy to contraception use was not significant (*β* = 0.057, *p* = 0.699). After controlling for contraception use and women’s autonomy, the association between emotional IPV and unintended pregnancy was decreased in magnitude and lost significance (p*ath c*^′^, *β* = 0.281, *p* = 0.094). Therefore, the effect of emotional IPV on unintended pregnancy was partially mediated by women’s autonomy but contraception use did not indicate any mediation effect in this relationship as *Path b*_1_ was not significant.

In the third mediation model (Fig. 2c, Table 4), IPV (physical, sexual, and/or emotional) was significantly negatively associated with women’s autonomy (*path a*_2_,* β* =  − 0.307, *p* = 0.009) and IPV was significantly positively associated with unintended pregnancy (*path c*, *β* = 0.332, *p* = 0.021). The negative association between women’s autonomy and unintended pregnancy, controlling for IPV, was also significant (*Path b*_2_,* β* =  − 0.537, *p* < 0.001). However, the associations between IPV with contraception use (*path a*_1_), contraception use with unintended pregnancy (*path b*_1)_, and women’s autonomy with contraception use were not significant. After controlling for women’s autonomy and contraception use, the coefficient for IPV was decreased in magnitude and significance (p*ath c*^′^, *β* = 0.300, *p* = 0.040). Therefore, the effect of IPV on unintended pregnancy was partially mediated by women’s autonomy, but not by contraception use.

In the final mediation model (Fig. 2d, Table 4), multiple partner control was significantly associated with contraceptive use (*path a*_1_,* β* =  − 0.391, *p* = 0.006) and women’s autonomy (*path a*_2_, *β* =  − 0.661, *p* < 0.001). When multiple partner control was adjusted in the model, women’s autonomy (*Path b*_2_, *β* =  − 0.519, *p* < 0.001), but not contraception use (*Path b*_1_,* β* =  − 0.259, *p* = 0.113), was significantly associated with unintended pregnancy. After controlling for contraception use and women’s autonomy, the coefficient for multiple partner control decreased in magnitude from *path c*, *β* = 0.453 to *path c*^′^, *β* = 0.365 and reduced in significance from *p* = 0.004 to *p* = 0.022, indicating partial mediation of the effects of multiple partner control on unintended pregnancy by women’s decision-making autonomy. Again, despite *path a*_1_ was significant and *path c*′was reduced in both magnitude and significance, contraceptive use did not meet established mediation criteria because *path b*_1_ was not significant.

Overall, in the multivariate logistic mediation analysis, three partial mediation effects were identified. The first effect was the association between emotional IPV and unintended pregnancy, which was partially mediated by women’s decision-making autonomy after controlling for potential confounders. Accordingly, the direct, indirect effect through women’s autonomy, total indirect effect, and total effects of emotional IPV on unintended pregnancy were (AOR 1.32, 95% CI: 0.95, 1.84), (AOR 1.23, 95% CI: 1.04, 1.46), (AOR 1.37, 95% CI: 1.08, 1.74), and (AOR 1.81, 95% CI: 1.26, 2.60) respectively. Therefore, the total proportion mediated was 52.8% and women’s autonomy alone mediated 34.5% of the total effect of emotional IPV on unintended pregnancy. The second effect was IPV (physical, sexual, and/or emotional) and unintended pregnancy, which was again partially mediated by women’s autonomy. Accordingly, the direct effect of IPV on unintended pregnancy was AOR 1.35 (95% CI: 1.01, 1.80), indirect effect through women’s autonomy was AOR 1.18 (95% CI: 1.01, 1.37), and total indirect effect was AOR 1.18 (95% CI: 1.01, 1.39). Therefore, about 35.4% of the total effect of IPV on unintended pregnancy was mediated by women’s autonomy. The third effect was the partial mediation effect of women’s autonomy in the association between multiple partner controlling behaviour and unintended pregnancy. In this model, the mediators mediated about 55% of the total effect of multiple partner controlling behaviours on unintended pregnancy, while women’s autonomy alone mediated 42.4% of the total effect (Table 5).

**Table 5:** The direct, indirect, and total effects of different forms of IPV on unintended pregnancy adjusting for mediators, Ethiopian Demographic and Health Survey, 2016

IPV forms	Effects	AOR (95% CI)^a^
Emotional IPV	Direct effect	1.32 (0.95, 1.84)
	Indirect effect through contraception use	1.11 (0.95, 1.31)
	Indirect effect through women’s autonomy	1.23 (1.04, 1.46)*
	Total indirect effect	1.37 (1.08, 1.74)*
	Total effect	1.81 (1.26, 2.60)**
	Total proportion mediated = 52.8%	
	Proportion mediated through women’s autonomy only = 34.5%	
IPV (physical, sexual, emotional)	Direct effect	1.35 (1.01, 1.80)*
	Indirect effect through contraception use	1.00 (0.93, 1.08)
	Indirect effect through women’s autonomy	1.18 (1.01, 1.37)*
	Total indirect effect	1.18 (1.01, 1.39)*
	Total effect	1.59 (1.17, 2.17)**
	Total proportion mediated = 35.6%	
	Proportion mediated through women’s autonomy only = 35.4%	
Multiple partner control behaviours	Direct effect	1.44 (1.06, 1.97)*
	Indirect effect through contraception use	1.11 (0.95, 1.29)
	Indirect effect through women’s autonomy	1.41 (1.10, 1.80)**
	Total indirect effect	1.56 (1.15, 2.11)**
	Total effect	2.25 (1.49, 3.38)***
	Total proportion mediated = 54.9%	
	Proportion mediated through women’s autonomy only = 43.4%	

*IPV* intimate partner violence, *AOR* adjusted odds ratio, *CI* confidence interval; ^a^The models were adjusted for age of women, education, rurality (urban/rural), religion, region of residence, number of children ever born, and wealth; ***p < 0.001, **p < 0.01, *p < 0.05

## Discussion

The current study investigated unintended pregnancy in relation to different forms of IPV in Ethiopia and the role contraceptive use and women’s autonomy plays in these relationships. We computed a series of multiple mediation logistic analyses to examine the interplay between the IPV types, contraceptive use, women’s autonomy, and unintended pregnancy. Women’s autonomy, but not contraceptive use, had a significant partial mediation role in the relationship of unintended pregnancy with some forms of IPV and unintended pregnancy. Given that unintended pregnancies are common in Ethiopia and women have less power in intimate relationships, this study provides insights into the need to develop and initiate culturally appropriate women’s empowerment interventions in maternal health programs to mitigate some of the negative reproductive health impacts of IPV.

Women’s experience of IPV (physical, sexual, and/or emotional) was associated with unintended pregnancy, which is in line with previous research that investigated the association between combined forms of IPV with unintended pregnancy [11–13, 35, 50]. Though our study was cross-sectional, which limits our ability to draw conclusions regarding causality, the effect of IPV on unintended pregnancy was both direct and indirect. The direct effect could be through coerced pregnancy (coercion by husband to become pregnant) or coerced unprotected sex (coercion by partner to have sex against her will) [41, 42]. This study further revealed that IPV might influence unintended pregnancy indirectly by reducing women’s autonomy. This could be because abusive partners might dominate women economically and emotionally that may cause women’s inability to make decisions freely [32].

In this study, contraception use did not show any mediation role in the relationship between IPV and unintended pregnancy. A similar finding from the U.S also revealed that the significant association between IPV and unintended pregnancy, where abused women were twice as likely as non-abused women to have had an unintended pregnancy, was not mediated by condom use. In this study, condom use had a positive association with unintended pregnancy and IPV had a negative association with condom use but both associations were not significant [51]. While some previous evidence has shown that IPV is associated with contraception use [18–20], in this study the association between IPV and contraception use was not significant. In Ethiopia, contraceptive use is generally low; for example, in this sample, only 24% of women were using contraception. Women in an abusive relationship share similar other socio-cultural and religious factors that hinders Ethiopian women from contraception access and use such as religious objection, community disapproval, rumours and perceived side effects among others [37]. As a result, there may be other immediate factors influencing women’s contraception use.

This study revealed that emotional IPV was negatively associated with women’s autonomy and women’s autonomy, in turn, was negatively associated with unintended pregnancy. However, there was no direct association between emotional IPV and unintended pregnancy after adjusting for women’s autonomy. This implies that the association between emotional IPV and unintended pregnancy was explained by the role of decision-making autonomy as mediator. Women who experienced emotional abuse could have reduced control over their reproductive choices and, potentially, reduced access to resources to achieve this. There is also evidence that emotional IPV is often accompanied by other forms of IPV [2] and the synergistic effect of these co-occurrences might lead to the strong association between emotional IPV and low women’s autonomy.

In this study, multiple partner controlling behaviour was significantly associated with unintended pregnancy, low contraceptive use, and low women’s autonomy. Simultaneously, women’s autonomy was associated with unintended pregnancy suggesting that not only partner control and unintended pregnancy were associated, but also partner control may influence unintended pregnancy by reducing women’s autonomy. Partner controlling behaviour is a reflection of power dynamics in an intimate relationship and husbands’ attempt to closely control and monitor their wives’ behaviour may affect women’s autonomy, contraceptive access and use, and fertility control [15, 27, 40–42]. Our finding demonstrated that the higher the number of partner controlling behaviours, the more severely a woman was being controlled; therefore, her autonomy in decision-making is lower, and her ability to control her fertility is more likely to be compromised compared with women not subjected to controlling behaviours. In Ethiopia, where patriarchal views are common, controlling behaviour is considered an acceptable behaviour for husbands in interactions with their wives [52, 53]. For this reason, women who have not experienced any partner control and those who experienced single partner control might not differ significantly in terms of the impact of partner control on their reproductive intentions.

The current study showed that physical IPV was associated with women’s autonomy, but not with contraceptive use nor unintended pregnancy. It is reasonable to think that women for fear of physical abuse might not have overall freedom and might therefore refrain from making their own decisions. The lack of a significant association between physical abuse and unintended pregnancy is supported by some previous research [35, 44] but contradicts findings from other studies [11, 50, 54]. In contrast, sexual IPV was significantly associated with unintended pregnancy but sexual IPV did not show a significant association with contraceptive use and women’s autonomy. This implies that the association between sexual IPV and unintended pregnancy was direct and could be because of forced unprotected sex despite risk of pregnancy during the ‘unsafe period of conception’ [41].

In this study, a lack of autonomy was strongly associated with high odds of unintended pregnancy in all the models. The more women have autonomy, the more likely they are to have access to and control over resources, access to health care, and the ability to decide on fertility (how many children to have and when to have the children) [32] thereby protecting them from unintended pregnancy. Contrary to the general perception, the association between contraception use and unintended pregnancy was not significant. The majority of women in the sample (76%) were not utilizing contraception and we assume that this high non-use may have affected the results. Moreover, further is required to understand what types of contraception were used, how effective these contraceptives were, and how consistently women have been using contraception. Lastly, while we hypothesized that there might be a relationship between the two mediators, that is, when women are autonomous, they are more likely to use contraception, our study did not show any significant association between these two variables. In this study, we have measured women’s overall autonomy in household matters and this might not necessarily reflect their autonomy in contraceptive choices.

As there is no prior study that has examined the role of IPV on unintended pregnancy and how women’s autonomy and contraception use plays a role in this relationship, the findings could contribute to design interventions for women in abusive relationships in Ethiopia that help mitigate the detrimental reproductive health effects of IPV. However, the findings should be considered in light of some limitations. Data for the current analysis were drawn solely from a cross-sectional study so causal inferences could not be made. Women with unintended pregnancies or low autonomy in decision-making may enter into violent relationships. Therefore, future prospective studies are needed to examine the temporal order of the IPV-unintended pregnancy association and potential mediators in this relationship. The partial mediation maintains that women’s autonomy accounts for some, but not all, of the relationship between some forms of IPV and unintended pregnancy. This implies that the assumed pathways are not entirely established. Another limitation is that although potential confounding variables were included, there could still be some residual confounding effects. Reports of IPV may also be underestimated due to social desirability bias. However, the study has strictly followed WHO strategies for domestic violence research which should minimize such under-reporting [55].

## Conclusions

This study has indicated that sexual IPV, emotional IPV, IPV (physical, sexual, and/or emotional), and multiple controlling behaviours were associated with unintended pregnancy. The associations of emotional IPV, IPV (physical, sexual, and/or emotional), and multiple controlling behaviours with unintended pregnancy were partially mediated by women’s autonomy where women’s autonomy mediated about 35, 35, and 43% of the total effect of these relationships, respectively. However, there was no mediation effect of contraception use in the relationships between these forms of IPV and unintended pregnancy. Reproductive health programs and strategies designed to improve fertility choices among women in Ethiopia should address both intra- and interpersonal factors in order to create conditions under which women are empowered to involve in making decisions about their own fertility. Interventions with women’s partners that reduce the incidence of IPV are also necessary. In the meantime, focussed interventions on improving victimized women’s decision-making power may help mitigate the effect of IPV on unintended pregnancy and other reproductive health problems.

## Data Availability

The datasets supporting the conclusions of this article are freely available to the public at www.measuredhs.com and can be accessed after request is made to and approved by the DHS program.

## Abbreviations

AOR: Adjusted odds ratio
CI: Confidence interval
COR: Crude odds ratio
DHS: Demographic and health survey
EDHS: Ethiopian demographic and health survey
IPV: Intimate partner violence
WHO: World Health Organization
